# Increased Regulatory T-Cell Activity and Enhanced T-Cell Homeostatic Signaling in Slow Progressing HIV-infected Children

**DOI:** 10.3389/fimmu.2019.00213

**Published:** 2019-02-12

**Authors:** Julia Roider, Abigail Ngoepe, Maximilian Muenchhoff, Emily Adland, Andreas Groll, Thumbi Ndung'u, Henrik Kløverpris, Philip Goulder, Alasdair Leslie

**Affiliations:** ^1^Africa Health Research Institute, University of KwaZulu-Natal, Durban, South Africa; ^2^Department of Paediatrics, Peter Medawar Building for Pathogen Research, Oxford University, Oxford, United Kingdom; ^3^HIV Pathogenesis Programme, Doris Duke Medical Research Institute, University of KwaZulu-Natal, Durban, South Africa; ^4^Department of Infectious Diseases, Medizinische Klinik IV, Ludwig-Maximilians-University Munich, Munich, Germany; ^5^Department of Virology, Max von Pettenkofer Institute, Ludwig-Maximilians-University Munich, Munich, Germany; ^6^German Center for Infection Research (DZIF), Partner Site Munich, Munich, Germany; ^7^Faculty of Statistics, TU Dortmund University, Dortmund, Germany; ^8^Ragon Institute of Massachusetts General Hospital, Massachusetts Institute of Technology and Harvard University, Cambridge, MA, United States; ^9^Max Planck Institute for Infection Biology, Berlin, Germany; ^10^Department of Infection and Immunity, University College London, London, United Kingdom; ^11^Department of Immunology and Microbiology, University of Copenhagen, Copenhagen, Denmark

**Keywords:** pediatric HIV-infection, regulatory T cells (Treg), immune activation (IA), IL-7, homeostatic signaling, pediatric slow progression, IL-10, immune regulation

## Abstract

Pediatric slow progressors (PSP) are rare ART-naïve, HIV-infected children who maintain high CD4 T-cell counts and low immune activation despite persistently high viral loads. Using a well-defined cohort of PSP, we investigated the role of regulatory T-cells (T_REG_) and of IL-7 homeostatic signaling in maintaining normal-for-age CD4 counts in these individuals. Compared to children with progressive disease, PSP had greater absolute numbers of T_REG_, skewed toward functionally suppressive phenotypes. As with immune activation, overall T-cell proliferation was lower in PSP, but was uniquely higher in central memory T_REG_ (CM T_REG_), indicating active engagement of this subset. Furthermore, PSP secreted higher levels of the immunosuppressive cytokine IL-10 than children who progressed. The frequency of suppressive T_REG_, CM T_REG_ proliferation, and IL-10 production were all lower in PSP who go on to progress at a later time-point, supporting the importance of an active T_REG_ response in preventing disease progression. In addition, we find that IL-7 homeostatic signaling is enhanced in PSP, both through preserved surface IL-7receptor (CD127) expression on central memory T-cells and increased plasma levels of soluble IL-7receptor, which enhances the bioactivity of IL-7. Combined analysis, using a LASSO modeling approach, indicates that both T_REG_ activity and homeostatic T-cell signaling make independent contributions to the preservation of CD4 T-cells in HIV-infected children_._ Together, these data demonstrate that maintenance of normal-for-age CD4 counts in PSP is an active process, which requires both suppression of immune activation through functional T_REG_, and enhanced T-cell homeostatic signaling.

## Introduction

In the absence of antiretroviral therapy (ART), HIV-infected children typically progress rapidly to AIDS (1). “Pediatric slow progressors” (PSP) are a subgroup of vertically HIV-infected children who maintain their CD4 counts despite being antiretroviral treatment-naive (2–7). These children share certain fundamental immunological characteristics with the natural hosts of nonpathogenic SIV infection such as sooty mangabeys, including low immune activation on CD4 T-cells in the face of persistent high viral loads (7–10). Here we investigate the role of immune regulation by regulatory T-cells (T_REG_) and of IL-7-mediated T-cell homeostasis, in maintaining this beneficial phenotype in a well-described cohort of PSP (7).

Persistent immune activation is a hallmark of HIV infection in humans and is an important driver of disease progression that is independent of viral load (11–13). Indeed, in pediatric HIV infection, it is CD4 T-cell activation and not viral load that is the strongest predictor of CD4 decline (7, 13, 14). In the non-human primate model of HIV infection, the rapid resolution of immune activation during acute infection also distinguishes non-pathogenic infection of SIV in sooty mangabeys from pathogenic infection in rhesus macaques (15). In sooty mangabeys, this resolution of immune activation is associated with an up-regulation of the canonical T_REG_ markers CD25 and FoxP3 on CD4 and CD8 T-cells (16), and increased production of the key regulatory cytokine IL-10 (17). Moreover, T_REG_ numbers in sooty mangabeys remain stable, whilst they are severely depleted in rhesus macaques, in correlation with disease state (18). The importance of T_REG_ in pediatric non-progression has not been assessed to date, but the similarities between PSP and non-progressive SIV infection suggest a possible role for these cells.

T_REG_ are required to prevent autoimmunity and to regulate over-reactive immunity in the setting of chronic infections, including HIV infection (19–21). They can exert their suppressive function through cell-cell contact (22, 23), disruption of metabolic pathways (24), or by releasing soluble suppressive factors like IL-10 or TGF-b (25, 26) [reviewed in Yamaguchi et al. (27)]. T_REG_ make up ~1% of CD4 T cells in untreated HIV-infected adults, but are more frequent in early life, and can constitute up to 15% of total CD4 T-cells in the fetus (28). Their frequency is inversely correlated with HIV-driven immune activation (14, 29–34), and a decline in absolute T_REG_ numbers coincides with disease progression (14, 33, 35–38). In addition, T_REG_ have been shown to be comprised of phenotypically and functionally diverse subsets. Miyara et al. identified three phenotypically distinct subsets that differed in their suppressive capacity: CD45RA^+^FoxP3^lo^ resting T_REG_ cells and CD45RA^−^FoxP3^hi^ activated T_REG_ cells, both of which were suppressive *in vitro*, and a cytokine-secreting CD45RA^−^FoxP3^lo^ subset that did not display suppressive activity *in vitro*. Terminally differentiated activated T_REG_ cells rapidly died whereas resting T_REG_ cells proliferated and converted into activated T_REG_ cells *in vitro* and *in vivo* (39).

In addition to T_REG_ activity, non-progression in sooty mangabeys has been linked to preservation of IL-7 signaling in T-cells (40). This pleiotropic cytokine is crucial for the development and homeostasis of T-cells, promotes antigen-specific expansion and memory formation (41–44), and can reverse T-cell exhaustion (45). Immune failure in both adult and pediatric HIV infection is associated with perturbations in IL-7 signaling (46, 47) and reduced responsiveness (48, 49). In adult long-term non-progressors, IL-7R (CD127) expression is preserved on central memory and effector memory CD4 T-cell compartments when compared to untreated progressors (50). Again, however, the importance of IL-7 to sustaining CD4 T-cell levels in PSP remains unknown.

In this study, we investigate two mechanisms by which PSP maintain their CD4 counts in the face of on-going viral replication: first, via strong regulatory T-cell responses that reduce immune activation; and, second, via intact IL-7 receptor signaling that preserves homeostatic proliferation. Both mechanisms are interlinked by the main driver of pathogenesis in HIV infection: chronic immune activation.

## Materials and Methods

### Study Participants

Peripheral blood mononuclear cells (PBMC) and plasma of vertically HIV-1 C clade-infected children and age matched healthy controls all from Southern Africa and predominantly of Zulu origin, were obtained from clinics in Durban, South Africa (Ithembalabantu Clinic and Prince Mshiyeni Hospital) (see Table 1 for cohort characteristics). In the present study, pediatric slow progressors (PSPN; *n* = 12) are defined as vertically HIV-infected, ART-naïve, CD4 count >450/mm^3^ at age >5 years. Pediatric future progressors (PFP; *n* = 11) were meeting inclusion criteria for PSPN at the time point used for the experiments but progressed in the longitudinal follow-up. Pediatric progressors (PP; *n* = 10) are defined here as vertically HIV-infected, ART-naïve, CD4 count <350/mm^3^ at age >5 years. This pediatric cohort has been followed up for over 5 years.

**Table 1:** Clinical characteristics of study cohort.

**Group**	***n***	**Age median (IQR)**	**CD4/mm^**3**^ median (IQR)**	**VL cp/ml median (IQR)**	**ART baseline**	**ART > baseline**
Pediatric slow progressors total (PSP)	23	10.2 (7–13.1)	752 (450–906)	37,600 (6,200–88,000)	no	no/yes
PSP subsequently on treatment/ Future progressors (FP)	11	12.4 (7–13.7)	704 (450–906)	59,000 (8000–130,000)	no	yes
PSP treatment naïve (PSPN)	12	9.85 (7.28–12.2)	796 (550–922.8)	24,000 (1015–57,000)	no	no
Pediatric progressors	10	10.65 (9–15.18)	233.5 (122.5–342.8)	120,000 (17,700–120,000)	no	yes
Pediatric healthy controls	20	13.15 (10–15.5)	N/A	N/A	N/A	N/A

*Overview of study subjects used for the different experiments in this study. Not for all shown subjects all experiments could be performed. All HIV-infected paediatric individuals were infected by vertical transmission*.

*N/A, not applicable.N/D, not done*.

Viral load measurement was performed using the COBRA AmpliPrep/COBAS TaqMan HIV-1 Test version 2.0 by Roche (CAP/CTMv2.0) (range, 20–10 millioncopies/ml) except at the Ithembalabantu Clinic where the BioMérieux NucliSens Version 2.0 Easy Q/Easy Mag (NucliSens v2.0) assay (range, 20–10 million copies/ml) was used.

Written informed consent was obtained for underage children from their caregivers. Additionally, assent to participate in the study was given directly by children in the appropriate age groups. Studies were approved by the University of the Free State Ethics Committee, Bloemfontein; Biomedical Research Ethics Committee, University of KwaZulu-Natal, Durban; and Research Ethics Committee, University of Oxford.

### Sample Processing—PBMC and Plasma

Plasma was separated by centrifugation and crypopreserved at −80°C. Peripheral blood mononuclear cells (PBMCs) were isolated by Ficoll density gradient centrifugation and stored in liquid nitrogen until use.

### Flow Cytometry and ICS Assays

PBMCs were stained with fluorescent monoclonal antibodies against markers previously associated with T_REG_ / immune activation /IL-7R (Supplementary Table 1). Briefly, cells were thawed and rested in R10 medium overnight at 37°C in 5% CO2. For phenotyping cells were washed in 15 ml of PBS, centrifuged at 500 g for 5 min and room temperature and the supernatant removed. Cells were resuspended in a volume of 25 μl with titrated concentrations of fluorochrome-conjugated monoclonal antibodies against cell surface markers and the L/D fixable blue dead stain (Thermo Fisher Scientific) as a viability marker (antibodies used are listed in Supplementary Table S1) and stained for 20 min in the dark. Cells were again washed x 2 in PBS and resuspended in Fix/Perm solution (ebioscience) for intracellular staining (Ki-67, FoxP3) and incubated for 20 min at 4°C, followed by washing and incubating 20 min in 20% Goat serum for Fc-receptor blockade. Afterwards, cells were incubated with intracellular antibodies for 20 min at room temperature in the dark, washed x 2 and the pellet was suspended in 2% paraformaldehyde in PBS. For intracellular cytokine staining assays cells were first stimulated with PMA/Ionomycin (at a final concentration of 4 × 10^−5^M) in the presence of anti-CD28 and anti-CD49 (1 mg/ml), Brefeldin A and Monensin (5 mg/ml) (BD biosciences), for 5 h, followed by surface staining and intracellular staining as above. Rainbow beads were run at every experiment and compensation adjusted to keep peaks identical to ensure longitudinal comparability of experiments. Flow cytometry acquisition was performed on a BD LSRFortessa within 5 h of staining and analyzed using FlowJo version 9.9.5.

### IL-7 and sCD127 Plasma Assays

Plasma levels of IL-7 and sCD127 were quantified using commercially available enzyme-linked immunosorbent assay kits (R&D Systems and Neobiolab, respectively) neat in duplicate. Results are expressed in picograms per millilitre for IL-7 and nanograms per millilitre for sCD127.

### Statistical Analyses

Most statistical analyses were undertaken using Prism GraphPad Software version 7.0. After confirming non-Gaussian distribution within the majority of the parameters analyzed, Mann–Whitney test was used to compare continuous factors between two groups and Kruskal-Wallis test to compare continuous factors between > two groups and Benjamini, Krieger and Yekutieli's procedure for false discovery rate to correct for multiple comparisons. For paired analyses, Wilcoxon test was used and for paired analyses at multiple time points 2-way ANOVA analyses. Bivariate correlation analyses were performed using the Spearman rank correlation method with exact permutation *P*-values calculated. All *P*-values are two-sided, and a *P*-value of <0.05 was considered significant. In scatterplots, median values, and interquartile range are indicated.

The influence of several predictor variables (*n* = 39) on the absolute number of CD4 T-cell counts was assessed by least absolute shrinkage and selection operator (LASSO) principle (51) on scaled covariates using the generalized linear model (GLM) glmnet package of the statistical software R as described before (7, 52). For this method, the optimal tuning parameter λ, which reflects the amount of penalization and, hence, controls variable selection, is determined via 5-fold cross-validation on the basis of the model's deviance. A final unregularized conventional GLM (post-LASSO) is fitted on the selected set of covariates using the R-function glm (Supplementary Figures 1A,B).

A correlation matrix was used to visualize Spearman's correlations between selected variables of the LASSO analysis, comprising absolute CD4 T-cell count; viral load; frequencies of HLA-DR, Ki-67, IL-7R expression on bulk CD4 and CD8 T-cells; HLA-DR and IL-7R expression on CM CD4 T-cells; Ki-67 expression on CM T_REG_; frequencies of T_REG_ T_EMRA_, T_REG_ EM, suppressive T_REG_, non-suppressive cytokine secreting T_REG_, CD25^−^CD127^−^CD4 T-cells; absolute count of T_REG_/μl; as well as IL-10 production of T_REG_ and CD4 T-cells upon PMA/Ionomycin stimulation. A data set of *n* = 26 vertically HIV-infected, ART-naïve children above 5 years of age for whom all measurements were available from the same study visit was used for analysis. Variables are grouped on the basis of principal components analysis using the R package correlogram.

## Results

### Pediatric Slow Progressors Display Low General Immune Activation and Proliferation

**Figure 1:**
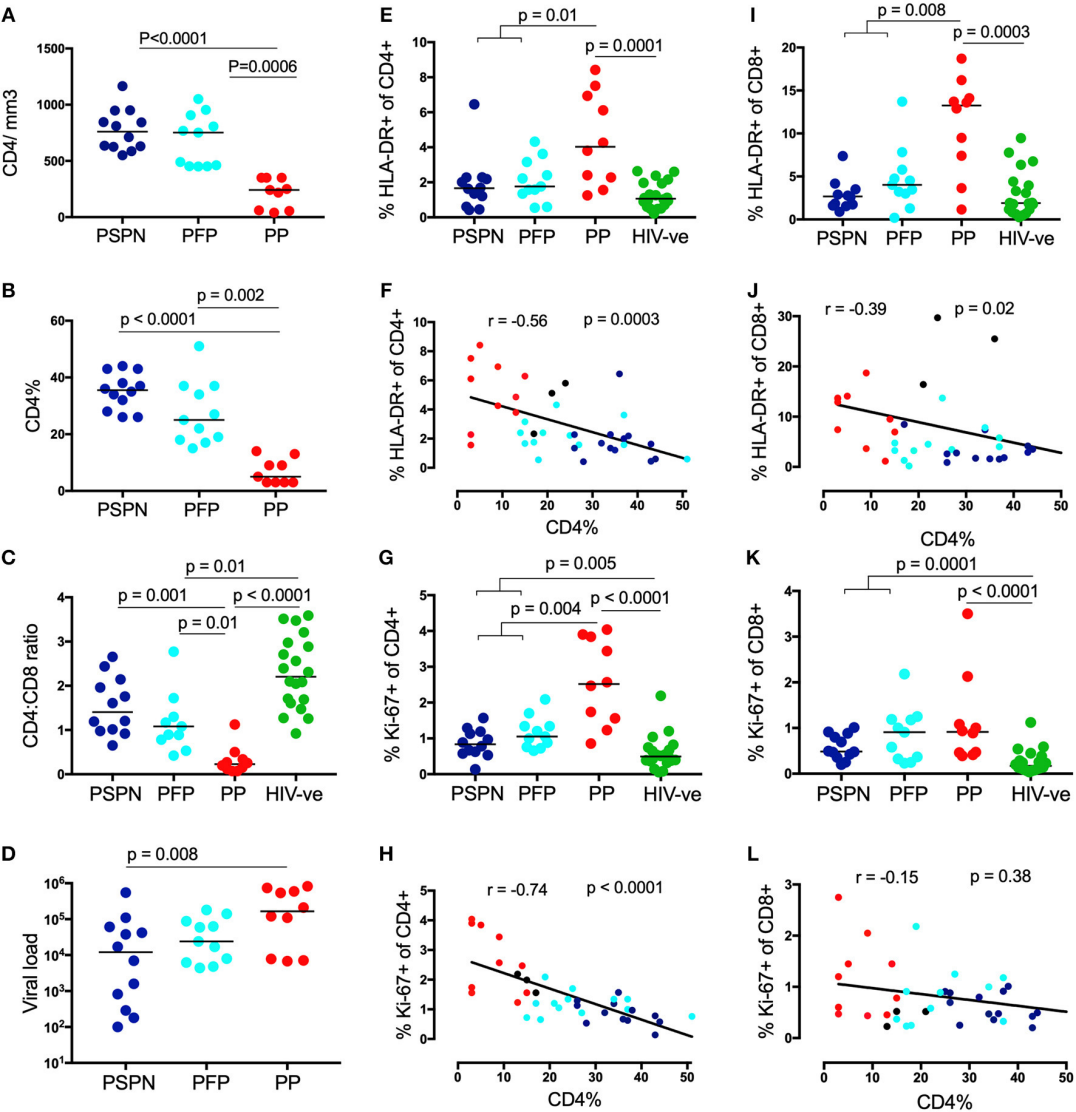
Pediatric slow progressors show low immune activation preferentially on CD4 T-cells. CD4 count absolute **(A)**, CD4 percentage **(B)**, viral load **(C)** in three pediatric groups: Pediatric slow progressors (PSP; *n* = 23) were defined here as treatment naïve, aged >5 years with CD4 counts >450/mm^3^ at the time point the experiments were performed. According to the longitudinal follow up we separated “pediatric future progressors” (PFP; *n* = 11; light blue) who would go onto treatment from true pediatric slow progressors, who stayed treatment naïve during the follow-up (PSPN; *n* = 12; dark blue). Pediatric progressors (PP; *n* = 10; red) were defined as treatment naïve, aged >5 years with CD4 counts <350/mm^3^. For CD4:CD8 ratio **(D)** age-matched uninfected controls (*n* = 20; green) were included as control group. **(E)** Frequency of HLA expression and its correlation with CD4 percentage **(F)** on CD4 T-cells within the 4 pediatric groups. (**G**,**H)**, same as **(E,F)** but for Ki-67 expression. **(I–L)** same as (**E–H)** but of CD8 T-cells. For scatterplots, median, and interquartile range are shown. Kruskal-Wallis test was performed and corrected for multiple comparisons. Only significant *p*-values (<0.05) are given. For correlations, Spearman ranks test was used. For correlations with CD4 percentage, all HIV-infected infants were used including those not fulfilling criteria for PSP nor PP (black dots; *n* = 3; CD4 count >350 and <450/mm^3^).

We defined pediatric slow progressors (PSP) as ART-naïve children aged >5years whose absolute CD4 count is >450 cells/mm^3^. To examine the T-cell correlates of low immune activation observed among PSP, we divided subjects into four study groups: (i) Pediatric slow progressors, who maintained CD4 counts at >450/mm^3^ throughout the study period and remained ART-naïve (PSPN; *n* = 12); (ii) Pediatric future progressors (PFP; *n* = 11): children who met the criteria to be categorized as PSP at baseline, but who later experienced CD4 decline to <350 cells/mm^3^ and commenced ART (iii) Pediatric progressors (PP; *n* = 10), defined as being ART-naïve with CD4 <350/mm^3^ who commenced treatment after the blood draw; (iv) Age-matched HIV-uninfected children (*n* = 20). The absolute CD4 counts, CD4 percentage, CD4:CD8 ratios and viral loads are shown in Figures 1A–D and clinical features summarized in Table 1. As previously reported (7), CD4 T-cell activation, measured by HLA-DR expression, was lower in both PSPN and PFP compared to PP and correlated with disease progression by CD4% (Figures 1E,F). CD4 T-cell proliferation, measured by Ki-67, also correlated very strongly with CD4%, but unlike HLA-DR, was significantly higher on PSPN and PFP compared to healthy controls (Figures 1G,H). The pattern of HLA-DR expression is similar on CD8 T-cells, but Ki-67 expression does not distinguish between progressors non-progressors (Figures 1I–L). These data in a subset of the pediatric cohorts previously described (7) confirm the previous findings showing low immune activation and, additionally, low proliferative activity on CD4 T-cells of PSP, both of which are strongly linked to CD4 count in ART-naïve children.

### Suppressive Regulatory T-Cells Are Increased in Pediatric Slow Progressors

**Figure 2:**
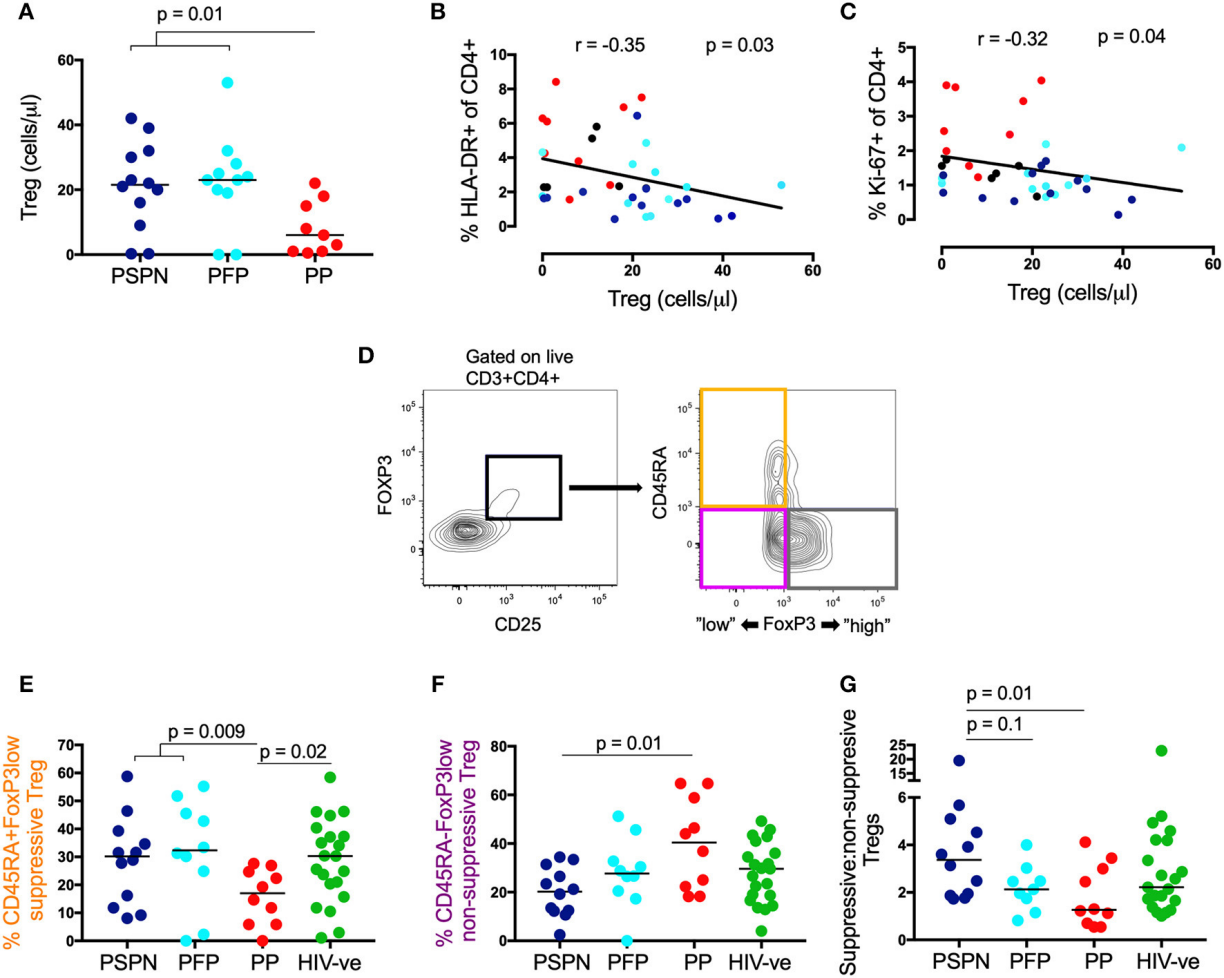
Suppressive regulatory T-cells are increased in pediatric slow progressors**. (A)** Absolute T_REG_ count/μl (gated on CD25^+^FoxP3^+^ CD4 T-cells) within the different pediatric groups: PSPN (dark blue; *n* = 12), PFP (light blue; *n* = 11), PP (red; *n* = 10) and **(B)** its correlation with HLA-DR and Ki-67 **(C)** on CD4 T-cells. For correlations all HIV-infected children were used including those not fulfilling criteria for PSP nor PP (black dots; *n* = 3; CD4 count >350 and <450/mm^3^). **(D)** Gating strategy for three functional T_REG_ (gated of CD25^+^FoxP3^+^ CD4 T-cells) memory subsets (39): (i) “activated”-suppressive T_REG_ (CD45RA^−^FoxP3^high^; gray); (ii) “resting”-suppressive T_REG_ (CD45RA^+^FoxP3^low^; orange) and cytokine-producing non-suppressive T_REG_ (CD45RA^−^FoxP3^low^; pink). **(E)** Same groups as **(A)** but including uninfected controls (green; *n* = 20) and showing the frequency of “resting”- suppressive T_REG_ (CD45RA^+^FoxP3^low^) and cytokine-producing non-suppressive T_REG_ (CD45RA^−^FoxP3^low^; **F)**. **(G)** Same as **(E,F)** but showing the ratio of all suppressive T_REG_ (CD45RA^+^FoxP3^low^ and CD45RA^−^FoxP3^high^; orange and gray population, respectively) to non-suppressive T_REG_ (CD45RA^−^FoxP3^low^; pink population) within the pediatric groups. For scatterplots, median, and interquartile range are shown. Kruskal-Wallis test was performed and corrected for multiple comparisons. For correlations, Spearman ranks test was used.

To determine whether T_REG_ play a role in controlling immune activation in these children, we studied CD4 T-cells co-expressing CD25 and the canonical transcription factor FoxP3 (39, 53) in the pediatric study groups described. The absolute number of T_REG_ was significantly elevated in both PSPN and PFP (Figure 2A), and this correlated negatively with HLA-DR and Ki-67 expression on CD4 T-cells (Figures 2B,C), consistent with a role for T_REG_ in preserving low immune activation in PSP. A similar trend was observed for T_REG_ frequency, although statistically not significant (Supplementary Figure 2).

To investigate whether these differences in T_REG_ numbers between the pediatric study groups might correspond with functional differences, we examined T_REG_ phenotype using expression levels of CD45RA and FoxP3 to identify three previously defined distinct functional T_REG_ subsets: (1) CD45RA+ve FoxP3^lo^ resting T_REG_ (Figure 2D; orange population), (2) CD45RA-ve FoxP3^hi^ activated T_REG_ cells (Figure 2D; gray population), both of which are suppressive *in vitro*; and (3) CD45RA-ve FoxP3^lo^ T_REG_ that secrete cytokines but have no direct immunosuppressive activity (Figure 2D; pink population) (39). No significant differences between the study groups were observed in the frequency of CD45RA-ve FoxP3^hi^ activated T_REG_ (data not shown). However, CD45RA+veFoxP3^lo^ resting T_REG_ (a functionally suppressive subset) were significantly higher in PSP compared to PP (Figure 2E), whilst non-suppressive, CD45-ve FoxP3^lo^ T_REG_, were highest in PP (Figure 2F). Together, the overall ratio of suppressive (combining the resting CD45+veFoxP3^lo^ and the activated CD45RA-ve FoxP3^hi^ subset): non-suppressive T_REG_ is higher in PSPN compared to both PP and PFP (Figure 2G). Overall these data suggest T_REG_ are both expanded and more functional in PSP compared to PP.

### Memory T_**REG**_ Proliferation and IL-10 Secretion Are Increased in Pediatric Slow Progressors

**Figure 3:**
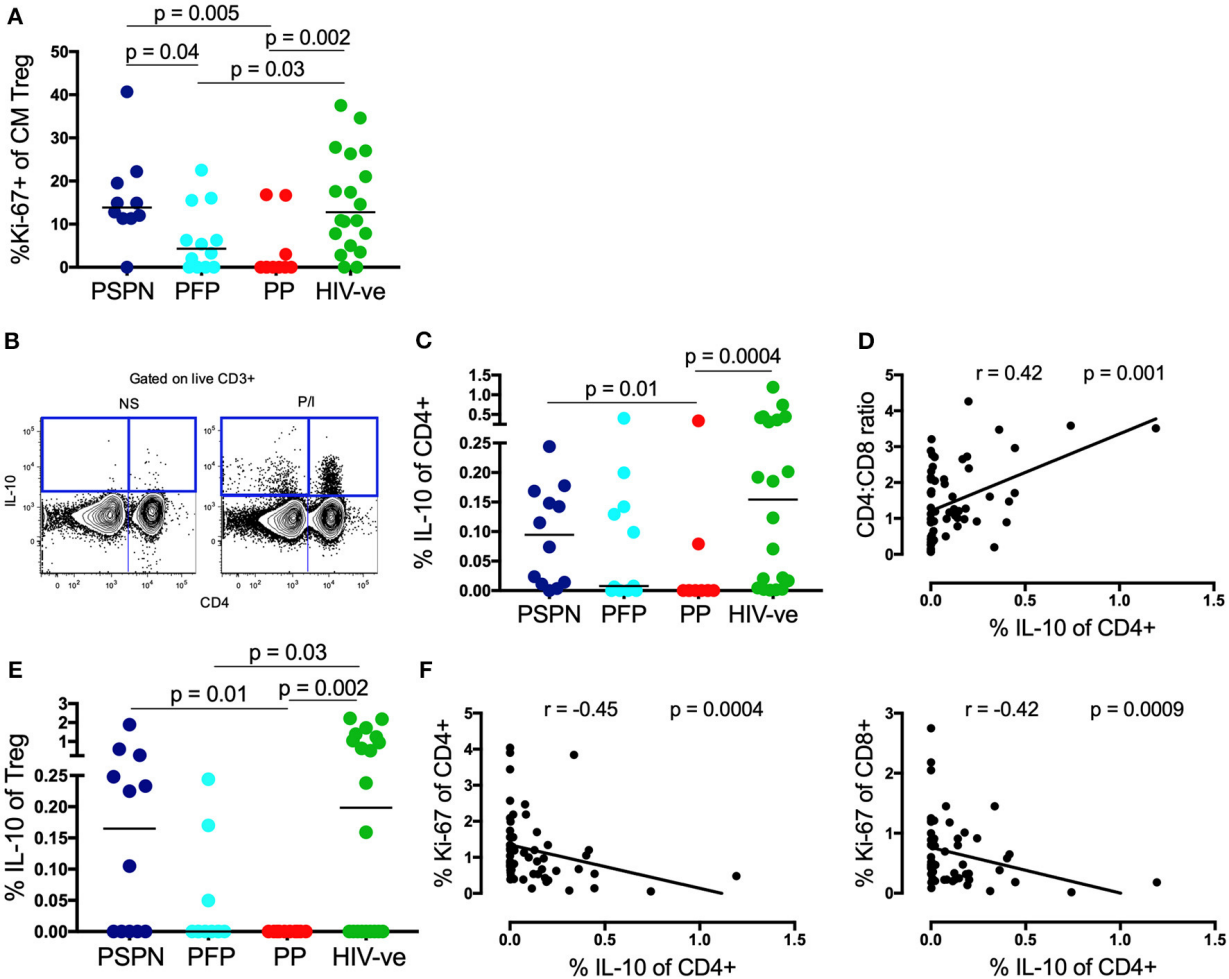
Memory T_REG_ proliferation and IL-10 secretion are increased in pediatric slow progressors. **(A)** Frequency of Ki-67+ve “central memory” T_REG_ (gated on CD4^+^CD25^+^FoxP3^+^CD45RA^−^CCR7^+^) within the pediatric groups: PSPN (dark blue; *n* = 12), PFP (light blue; *n* = 11), PP (red; *n* = 10), and uninfected controls (green; *n* = 20). **(B)** Exemplary FACS plot of one PSPN study subject showing CD4 (x-axis) vs. IL-10 (y-axis) staining (left: unstimulated control, right: stimulated with PMA/Ionomycin). **(C)** Same groups as **(A)** but showing IL-10 production of CD4 T-cells as measured by intracellular staining assay after 5 h stimulation with PMA/ Ionomycin and **(D**) its correlation with CD4:CD8 ratio within the whole pediatric cohort. **(E)** Same as **(C)** but of T_REG._ **(F)** Same as **(D)** but correlating IL-10 production of CD4 T-cells with Ki-67 expression on CD4 (left) and CD8 (right) T-cells. For correlations, all data available from pediatric subjects was used, including those not fitting criteria for PSP nor PP. For scatterplots, median, and interquartile range are shown. For correlations, Spearman ranks test was used. Kruskal-Wallis test was performed and corrected for multiple comparisons.

To further investigate T_REG_ activity, we next measured proliferation and production of the key anti-inflammatory cytokine, IL-10 (27). Much higher levels of *ex-vivo* proliferation, by Ki-67, were observed in the central memory T_REG_ subset of PSPN, compared to both PP and PFP (Figure 3A, see Supplementary Figure 3A for exemplary gating plots). Importantly, this was highly specific to central memory T_REG_, and not observed on central memory CD4 T-cells, on total T_REG_, or on total CD4s (Supplementary Figure 3B, Figure 1G). These data are consistent with the hypothesis that the expanded and phenotypically functional T_REG_ observed in PSP, are maintained through active, on-going proliferation in these subjects, and that this activity is required to reduce immune activation and prevent CD4 T-cell decline.

Evidence of this on-going immune regulation in PSP was also observed when we measured IL-10 production (Figure 3B). In response to non-specific stimulation with PMA/ionomycin, IL-10 production was severely impaired in PP, but preserved in PSPN (*p* = 0.01, Figure 3C). Again, PFP display an intermediate phenotype, and overall IL-10 production correlated with immune health, as measured by CD4:CD8 ratio (Figure 3D). As previously reported (54) IL-10 production by T_REG_ themselves was low, but remained elevated in PSPN compared to PP (Figure 3E). Overall, total IL-10 production by CD4 T-cells within all pediatric study subjects correlated inversely with Ki-67 expression in both bulk CD4 and CD8 T-cells (Figure 3F), consistent with a direct role for this cytokine in limiting immune activation. Taken together, these data suggest that the maintenance of CD4 T-cells in PSP is an active process, impairment of which, as in PFP, may precipitate disease progression.

### Increased IL-7/ sIL-7 Receptor in Pediatric Slow Progressors

**Figure 4:**
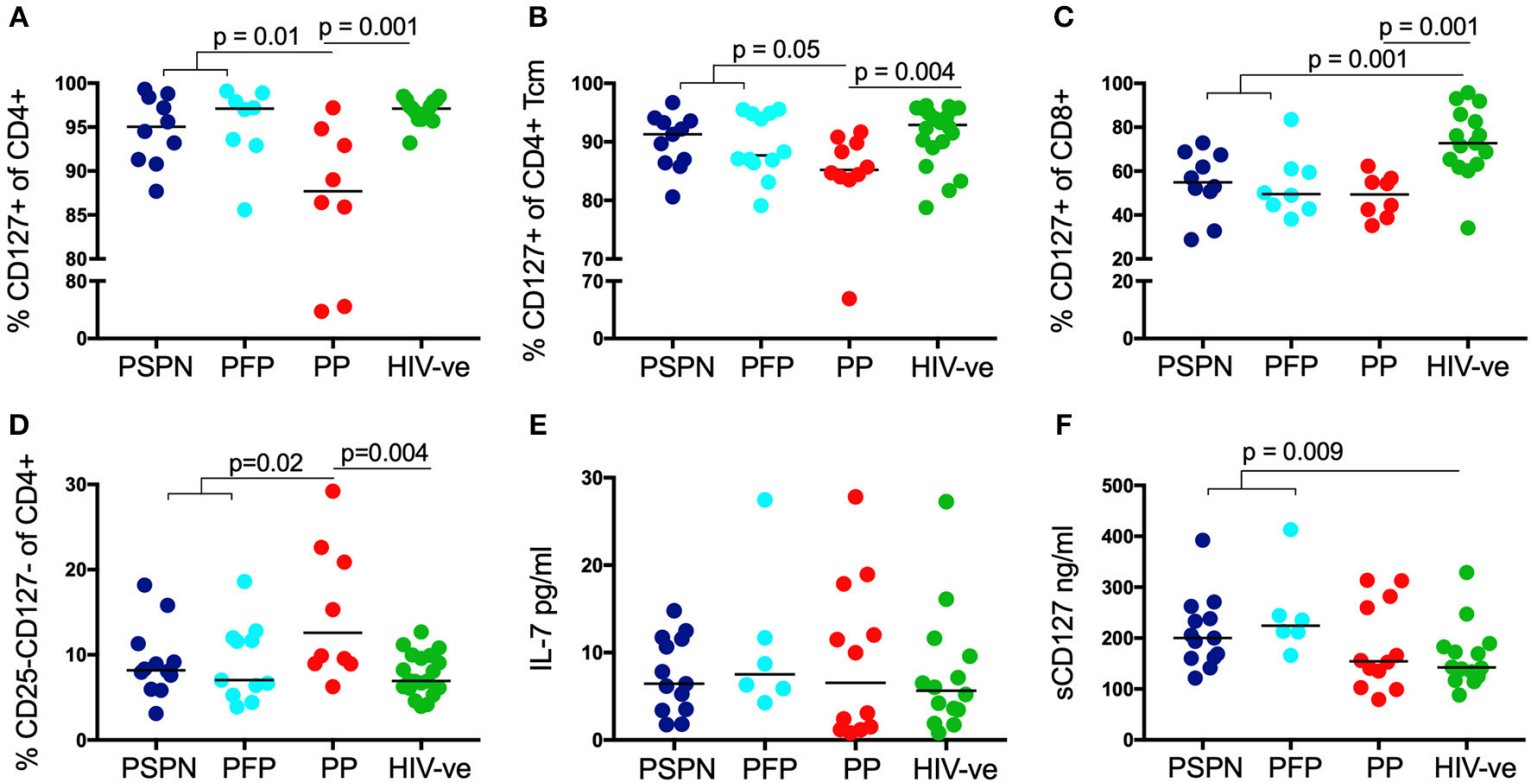
Increased IL-7/sIL-7 receptor in pediatric slow progressors. Frequency of IL-7R (CD127) expression on CD4 **(A)** and on central memory CD4 **(B)** T-cells in PSPN (dark blue; *n* = 10), PFP (light blue; *n* = 8), PP (red; *n* = 8), and uninfected pediatric controls (green; *n* = 15). **(C)** Same groups as before but showing frequency of IL-7R expression on CD8 T-cells and **(D)** frequency of CD4 T-cells double-negative for IL-2R (CD25) and IL-7R (CD127). **(E)** *Ex-vivo* plasma levels of IL-7 in pg/ml in PSPN (*n* = 13, dark blue), PFP (*n* = 6, light blue), PP (*n* = 12; red), and pediatric uninfected controls (*n* = 14; green). **(F)** Same as in **(E)** but showing plasma levels of IL-7R (sCD127) in ng/ml in the different groups. For scatterplots, median, and interquartile range are shown. Kruskal-Wallis test was performed and corrected for multiple comparisons.

Having established a role for immune regulation in PSP, we next examined the importance of homeostatic IL-7 signaling in these individuals. CD4 T-cell depletion in adult HIV infection is associated with a loss of the IL-7 receptor, CD127, whilst both non-progressing adults, and SIV-infected sooty mangabeys maintain high CD127 expression (40, 55, 56). Consistent with these data, we find CD127 is reduced on CD4 T-cells from PP compared to uninfected controls (*p* = 0.001) but preserved in both PSP groups (*p* = 0.01; Figure 4A). This is primarily a result of reduced expression on central memory T-cells (Figure 4B) and not in other memory subsets (Supplementary Figure 4). Interestingly, CD127 expression on CD8 T-cells is significantly lower in all HIV-infected individuals compared to healthy controls, irrespective of disease progression (Figure 4C). Furthermore, with respect to homeostatic T-cell proliferation mediated by IL-2 signaling via the high affinity IL-2 receptor CD25, we observed that CD25 CD127 double negative CD4 T-cells are expanded in PP compared to healthy controls, but not in PSP (Figure 4D). This finding, among HIV-infected children, is also consistent with the association in adults described between an expansion of CD4 T-cells lacking both CD25 and CD127 and progressive disease (55).

As CD4 T-cells are a main consumer of IL-7 *in vivo*, loss of these cells in progressive adult HIV infection has been linked with an increase in plasma levels of IL-7 (43). Thus, IL-7 itself is considered unlikely to be a limiting resource in HIV infection. However, recent data demonstrate that the bio-activity of IL-7 is directly enhanced by the presence of soluble CD127 (sCD127) (57). Indeed, the positive benefit of IL-7 therapy in SIV infected rhesus macaques was dependent on high levels of sCD127 (58). We therefore measured plasma levels of IL-7 and sCD127 in HIV-infected children. As expected, plasma IL-7 was not limiting in any of our designated groups, and indeed no significant differences were observed (Figure 4E). However, sCD127 levels were markedly higher in PSP (median 212 ng/ml) compared to both PP (154 ng/ml), and uninfected controls (142 ng/ml; *p* = 0.009). As reported elsewhere, plasma level of sIL-7R are far greater than that of IL7 itself, being in the 100–400 ng/ml range compared to 1–30 pg/ml for IL-7 [Figure 4F; (57, 59)]. Importantly, the fact they have significantly higher levels of sCD127 than healthy controls, again suggest that this is an active process in PSP, rather than a bystander effect of non-progression. Together with preserved expression of the surface receptor CD127, IL-7 signaling in PSP is likely to be enhanced by high levels of sIL-7R in plasma, to potentiate the homeostatic activity of IL-7, and preserve CD4 counts.

### Preservation of IL-7Ra and IL-2Ra Expression on CD4 T-Cells and T_**REG**_ Count Are Independent From Immune Activation and Strong Associates for Slow Progression in Pediatric HIV Infection

To determine the independent contribution of the multiple, often correlated parameters (see correlogram, Supplementary Figure 5) described to the maintenance of normal-for-age CD4 T-cell counts, we assessed the influence of each using a generalized linear model (GLM), applying the least absolute shrinkage and selection operator (LASSO) principle on scaled covariates as described before (Table 2) (7). In agreement with this previous study, a primary driver of slow progression in this current analysis is low immune activation on CD4 T-cells. However, Ki-67 expression on CD4 T-cells, which was not included in the previous analysis, supersedes HLA-DR expression on CM CD4 T-cells, previously identified as the strongest predictor of CD4 count. In addition to these markers of immune activation, the LASSO model, also identifies IL-7R (CD127) and IL-2R (CD25) expression on CD4 T-cells, IL7-R of CD8 T-cells, as well as absolute T_REG_ count and phenotype, as making independent contributions on CD4 count. Taken together, these data strongly support an important role for an active T_REG_ response and T-cell homeostatic signaling in maintaining normal-for-age CD4 counts in PSP.

**Table 2:** Association of immunological parameters with CD4 T-cell count.

**Variable**	**Lasso estimate**	**Post-lasso estimate**	***p*-value**
CD4+Ki67+	−0.180	−0.265	< 0.0001
CD4+CM HLA-DR+	−0.033	−0.156	< 0.0001
CD4+CD25-CD127-	−0.095	−0.117	< 0.0001
Treg count	0.069	0.111	< 0.0001
CD8+CD127+	0.032	0.063	< 0.0001
Treg EM	−0.026	−0.057	< 0.0001
Viral load	−0.007	−0.046	0.0002
Non-suppr Treg	−0.003	−0.044	0.002
CD4+HLA-DR+	−0.059	−0.025	0.133

*Standardized regression parameter estimates for the selected set of covariates by an unregularized conventional GLM (post-LASSO) using the R-function glm are shown in the middle two columns. In the right column the calculated p-value is shown. However, it has to be mentioned that the p-values and significance statements obtained from the post-LASSO analysis should be regarded with caution. In general, these values tend to underestimate the overall level of uncertainty in the estimates, as the uncertainty from the preceding LASSO procedure is ignored. Following covariates were included into the LASSO analysis but not selected and, hence, are not displayed in the table: Age, CD8^+^HLA-DR^+^, CD8^+^Ki-67^+^, T_REG_ frequency, T_REG_ memory subsets other than EM, T_REG_Ki-67^+^, T_REG_ EmraKi-67^+^, CD4^+^IL-10, and T_REG_ IL-10 production in response to PMA/Ionomycin, CD4^+^CD127^+^, CD4^+^ memory subsets CD127^+^, HLA-DR^+^ and Ki-67^+^, suppressive-activated T_REG_, suppressive-resting T_REG_, plasma IL-7, and sCD127.Selected regression parameter estimates with LASSO approach implemented in the R package glmnet are shown in the left column in HIV-infected children (n = 26) with available data for all regarded parameters*.

## Discussion

In this study, we investigate the underlying mechanisms of CD4 preservation in a well-defined cohort of HIV-infected pediatric slow progressors (PSP). These children share fundamental characteristics with the natural hosts of non-pathogenic SIV infection, most importantly the preservation of circulating CD4 T-cells, and the lack of immune activation, despite on-going viral replication. Here we find strong evidence that the maintenance of this non-progressive state is an active process, requiring both immune regulation via T_REG_ activity and IL-10 production, and enhancement of homeostatic IL-7 signaling. Notably, we also observe differences in phenotype and T_REG_ activity between PSP who remain stable through the course of study (PSPN) and those who go onto progress at a later time-point (PFP). This suggests that T_REG_ activity is particularly important in preventing progression, and that loss of this key function will precipitate development of disease. Moreover, this observation strengthens the argument that active involvement of T_REG_ is required to maintain non-progression in PSP, rather being a consequence of a preserved CD4 T-cell compartment.

The role of regulatory T-cells in HIV infection has been controversial, with some studies suggesting they are detrimental as they inhibit antiviral T-cell responses (60–62), whilst others find a beneficial role through the reduction of immune activation (63–66) [reviewed in Phetsouphanh et al. (67)]. However, in general, preservation of T_REG_ has been associated with positive outcomes; including non-progression in HIV elite controllers (68, 69), adult viremic slow progressors (70) and natural hosts of SIV infection (71). Conversely, a loss of circulating T_REG_ in adult progressors inversely correlates with the activation and apoptosis of CD8 T-cells (72), and experimental depletion in SIV-infected natural hosts increases immune activation and viral replication (73). The importance of measuring absolute counts has been emphasized previously (14, 35), in studies finding that the relative frequency of T_REG_ was increased in all HIV-infected individuals, compared to controls, but that the absolute number was greatly decreased, with the exception of elite controllers. Taken together, these studies strongly suggest T_REG_ can play a beneficial role in limiting HIV disease progression.

Our hypothesis that activation of T_REG_ in PSP is an important indicator of their functional involvement in immune regulation is supported by previous work showing adult HIV-infected elite controllers (EC) and viremic long term non-progressors (LTNPs) have more activated T_REG_ than healthy controls (74). Moreover, similar to our PSP, these EC have preserved IL-10 production in contrast to the controls. IL-10 is an anti-inflammatory cytokine that is critically important in preventing inflammatory and autoimmune diseases and is produced by multiple cells of the adaptive immune system including CD4 T-cells and T_REG_ as well as by innate cells [reviewed in Kwon and Kaufmann (54)]. Not all studies have shown a positive effect of IL-10 in adult HIV infection (75, 76) due to its negative impact on HIV-specific T-cell proliferation and effector function. It is possible that IL-10 has different effects in adult and pediatric HIV infection, as cytotoxic T-cells are not thought to be important in PSP (77). However, other studies have shown a correlation between IL-10 production, low immune activation, and slow disease progression in adult HIV infection (78) and genetic polymorphisms in the IL-10 promoter that are associated with higher IL-10 production have been shown to attenuate CD4 T-cell loss in HIV-infected individuals (79–81).

In addition to immune regulation, a key observation of our study is the apparent preservation and augmentation of IL-7 signaling in PSP. Firstly, and in contrast to progressing children, IL-7 receptor expression (CD127) is preserved in PSP, especially within the CD4 T-cell compartment. This correlates with parameters of disease progression and is in accordance to various studies of adult and pediatric HIV infection (50, 82–86). Notably, differences in CD127 expression are most prominent within the central memory compartment, which has recently been shown to be particularly dependent on IL-7 signaling (87). Similar preservation of CD127 expression in the central memory compartment has been described in adult elite controllers and LTNP (50, 88). In these studies, CD127 expression was directly linked to viral load, which cannot be true in the case of PSP, who are all viremic, again highlighting the likely differences in mechanism underlying HIV non-progression in adults and children.

Second, and most unexpectedly, we found PSP had increased plasma levels of sCD127 compared to PP and healthy controls. The precise details of how sCD127 influences IL-7 signaling and T-cell homeostasis are unclear. Progressive adult HIV infection was linked to increased plasma levels of sCD127, hypothesized to inhibit IL-7 availability (59). However, lower levels of sCD127 have been observed by others in HIV-infected patients compared to healthy controls (89). Moreover, higher levels of sCD127 in plasma were associated with improved immune reconstitution in SIV infected rhesus macaques treated with recombinant IL-7 (58). The potentiating effect of sCD127 on IL-7 signaling is supported by mechanistic studies showing it enhances the bioactivity of IL-7 when the cytokine is limiting, as it is presumed to be the situation *in-vivo* (57, 59). Therefore, we hypothesize that increased plasma sCD127 leads to enhanced bioactivity of IL-7 in PSP and, along with preserved surface CD127 expression, improves maintenance of CD4 T-cells through IL-7 homeostatic signaling. The fact that sCD127 levels are higher in PSP than healthy controls, suggests this is an important mechanism in preserving CD4 T-cell levels in these rare PSP individuals.

One potential limitation of this study is the use of surface CD25 as a marker of T_REG_, as this molecule is also an activation marker, whose expression may be affected by HIV-driven immune activation (90, 91). However, we have previously examined CD25 in HIV-infected and uninfected children and did not observe a significant impact of HIV on overall CD25 expression levels on CD4 T-cells (92). Furthermore, as the PSP group displayed very low immune activation, it is unlikely to have caused elevated CD25 expression within this group. An additional limitation is the lack of *in vitro* data to directly determine T_REG_ activity (39). Unfortunately, we did not have sufficient samples remaining from this historical cohort to perform such assays. However, the fact that we have identified multiple independent measures of enhanced T_REG_ activity in PSP, including increased T_REG_ frequency and proliferation, T_REG_ skewing toward more suppressive phenotypes, and enhanced IL-10 production, strongly supports the existence of enhanced T_REG_ suppressive capacity in the unique subset of PSP.

In this study, we observed various mechanisms contributing to CD4 T-cell preservation shared between PSP and the natural hosts of SIV infection. Firstly, as observed in natural hosts (8, 10, 16), PSP had low T-cell immune activation and proliferation as measured by HLA-DR and Ki-67 expression despite viremia and this correlated inversely with parameters of disease progression. Secondly, PSP showed increased absolute T_REG_ numbers and this was strongly inversely associated with immune activation (18). Thirdly, both CD4 T-cells and T_REG_ of PSP secreted more IL-10 than those of progressors which fits with the observation that in African green monkeys, but not in rhesus macaques, SIV infection leads to expression of an anti-inflammatory profile including IL-10, and TGF-β secretion and an increase in T_REG_ (17). And lastly, both PSP and natural hosts have a low frequency of CD4 T-cells lacking both IL-7R and IL-2R (CD127 and CD25) (55). These CD127-veCD25-ve CD4 T-cells show features of activated effectors and could therefore be key promoters of the overall level of immune activation and disease progression (55). LASSO modeling suggests that, although some of these parameters maybe linked through immune activation, both T_REG_ activity and T-cell homeostasis are independently involved in maintaining CD4 counts in PSP.

Over all, our findings support a model where PSP preserve their CD4 T-cells through actively maintaining low immune activation, requiring both immune regulation via T_REG_ activity and IL-10 production, and enhancement of T-cell homeostatic IL-7 signaling.

## Author Contributions

JR designed the study, conducted experimental work within the study, analyzed the data, and wrote the paper. AN, MM, and EA conducted experimental work within the study. AG performed statistical analyses within the study. TN supervised experimental work within the study. HK, PG, and AL supervised experimental work within the study, analyzed data, and wrote the paper. PG established research cohorts.

### Conflict of Interest Statement

The authors declare that the research was conducted in the absence of any commercial or financial relationships that could be construed as a potential conflict of interest.

## Supplementary Material

Supplementary Figure 1 **(A)** LASSO coefficient paths vs. tuning parameter λ and **(B)** deviance of the model via 5-fold cross-validation.

Supplementary Figure 2 Frequency of T_REG_ (%CD25^+^FoxP3^+^ of CD4 T-cells) in PSPN (dark blue; *n* = 12), PFP (light blue; *n* = 11), PP (red; *n* = 10) and uninfected pediatric controls (green; *n* = 20).

Supplementary Figure 3 **(A)** Exemplary gating strategy for Ki-67 expression of “central memory” TREG (CD4^+^CD25^+^FoxP3^+^CD45RA^−^CCR7^+^) in one PSPN (blue; top row) and one PP child (red; bottom row). **(B)** Frequency of Ki-67 expression on all TREG (gated on CD4^+^CD25^+^FoxP3^+^; left), of all central memory CD4 T-cells (CD45RA-CCR7^+^; CM; middle) and of Ki-67 expression on all CM T-cells (CD45RA^−^CCR7^+^; right) within the different pediatric groups: PSPN (dark blue; *n* = 12), PFP (light blue; *n* = 11), PP (red; *n* = 10) and uninfected pediatric controls (green; *n* = 20). Kruskal-Wallis test was performed and corrected for multiple comparisons.

Supplementary Figure 4 Frequency of IL-7R expression on naïve (TN; left), effector memory (EM; middle) and terminally differentiated (Temra; right) CD4 T-cells in PSPN (dark blue; *n* = 12), PFP (light blue; *n* = 11), PP (red; *n* = 10) and uninfected pediatric controls (green; *n* = 20). For scatterplots, median and interquartile range are shown. Kruskal-Wallis test was performed and corrected for multiple comparisons.

Supplementary Figure 5 Correlation matrix in ART-naïve HIV-infected children (*n* = 25) with available data for all regarded parameters. Positive correlations are colored in blue and inverse correlations in red with deeper color shading and bigger circle size reflecting stronger r-values. Correlations with *p* > 0.05 are left blank.

Supplementary Table 1 List of antibodies for flow cytometry.
