# Supplementary figures and images for: Increased Regulatory T-Cell Activity and Enhanced T-Cell Homeostatic Signaling in Slow Progressing HIV-infected Children
Source: Front Immunol. 2019 Feb 12;10:213. doi: 10.3389/fimmu.2019.00213 (PMC6379343; doi:10.3389/fimmu.2019.00213)

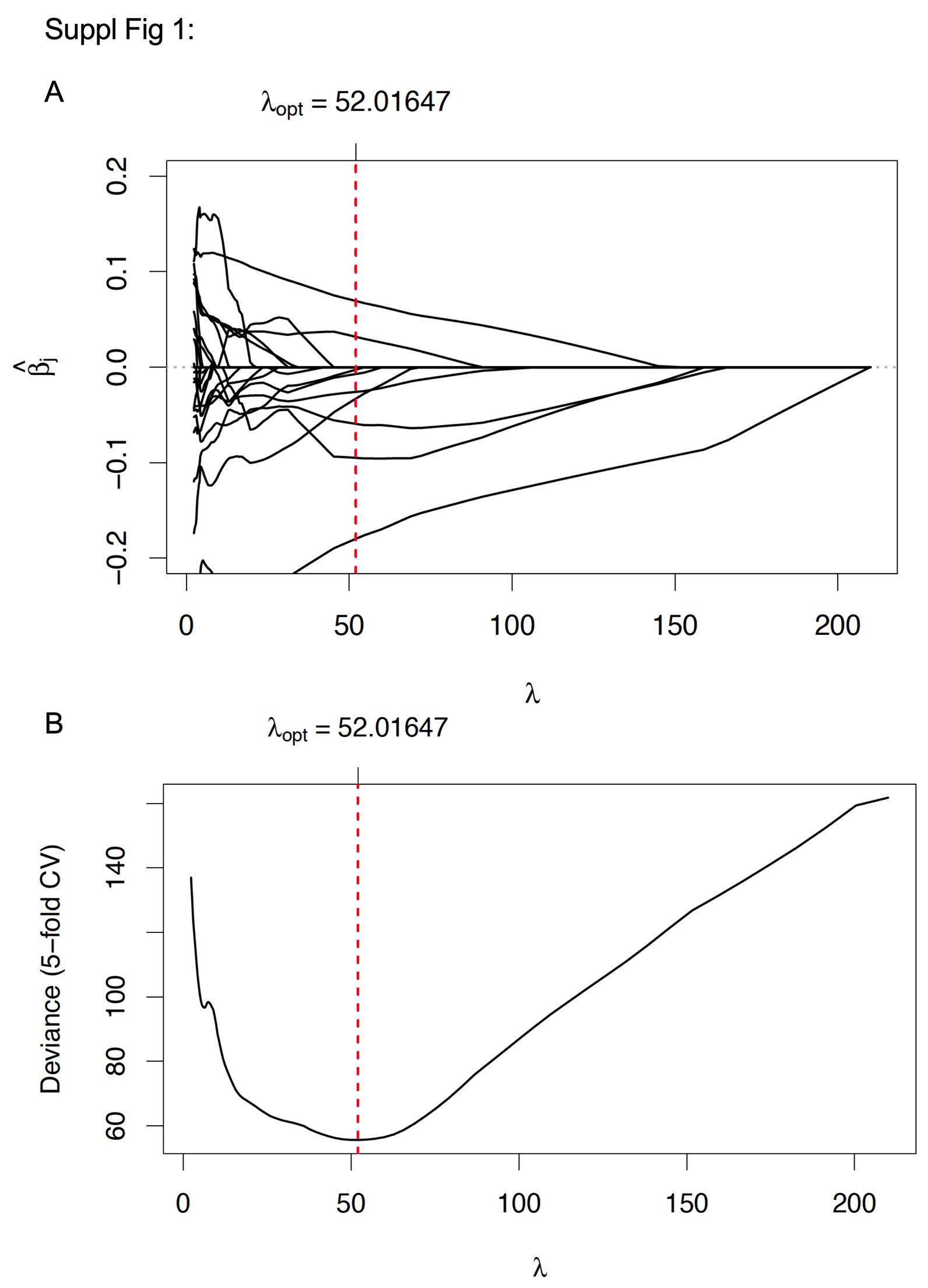

Supplement: Supplementary Figure 1 — (A) LASSO coefficient paths vs. tuning parameter λ and (B) deviance of the model via 5-fold cross-validation. [file Image_1.TIFF]

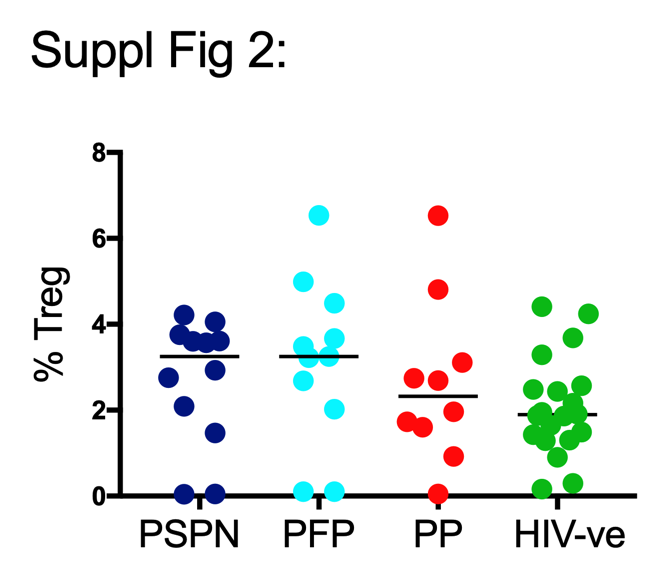

Supplement: Supplementary Figure 2 — Frequency of TREG (%CD25+FoxP3+ of CD4 T-cells) in PSPN (dark blue; n = 12), PFP (light blue; n = 11), PP (red; n = 10) and uninfected pediatric controls (green; n = 20). [file Image_2.TIFF]

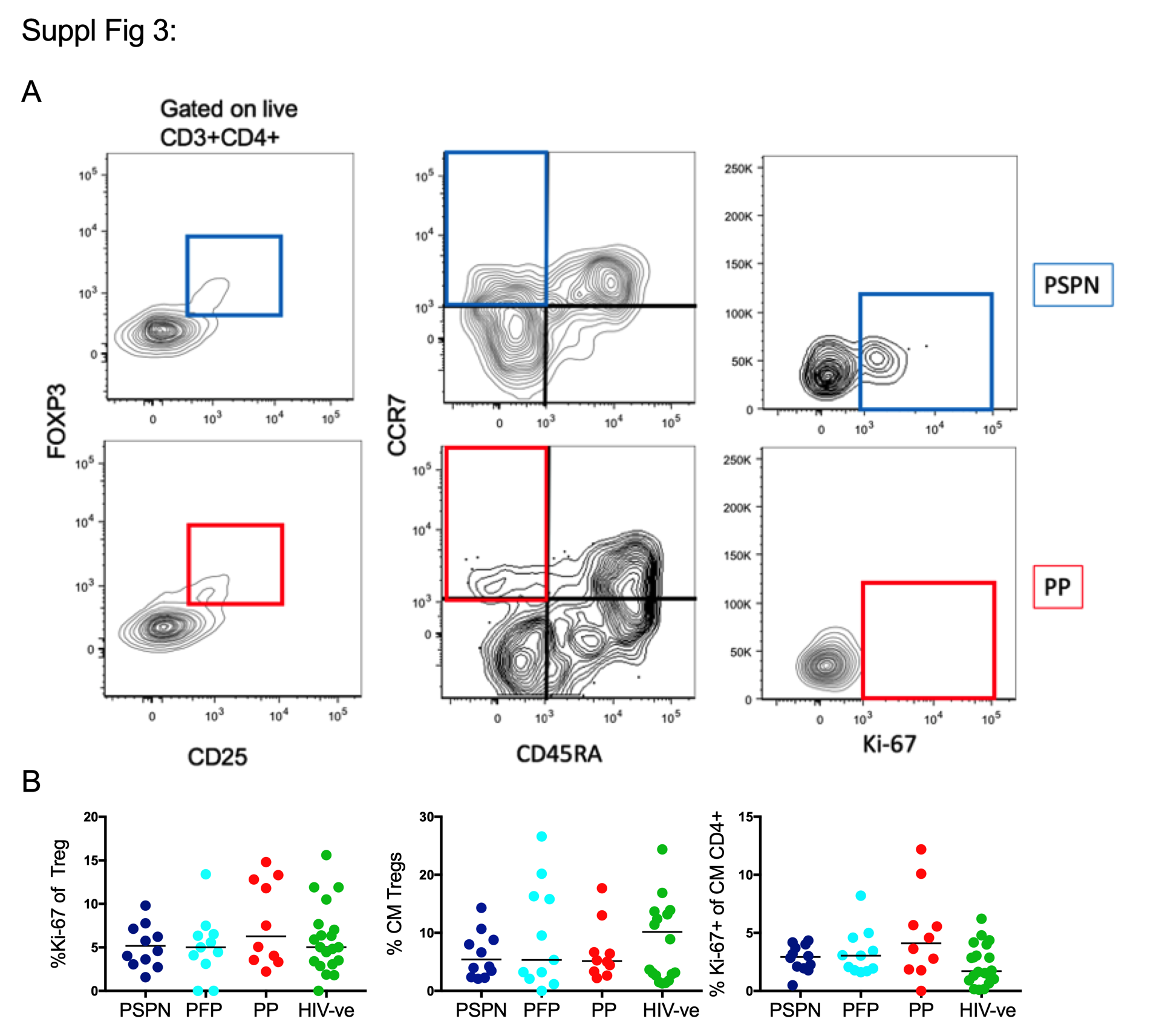

Supplement: Supplementary Figure 3 — (A) Exemplary gating strategy for Ki-67 expression of “central memory” TREG (CD4+CD25+FoxP3+CD45RA−CCR7+) in one PSPN (blue; top row) and one PP child (red; bottom row). (B) Frequency of Ki-67 expression on all TREG (gated on CD4+CD25+FoxP3+; left), of all central memory CD4 T-cells (CD45RA-CCR7+; CM; middle) and of Ki-67 expression on all CM T-cells (CD45RA−CCR7+; right) within the different pediatric groups: PSPN (dark blue; n = 12), PFP (light blue; n = 11), PP (red; n = 10) and uninfected pediatric controls (green; n = 20). Kruskal-Wallis test was performed and corrected for multiple comparisons. [file Image_3.TIFF]

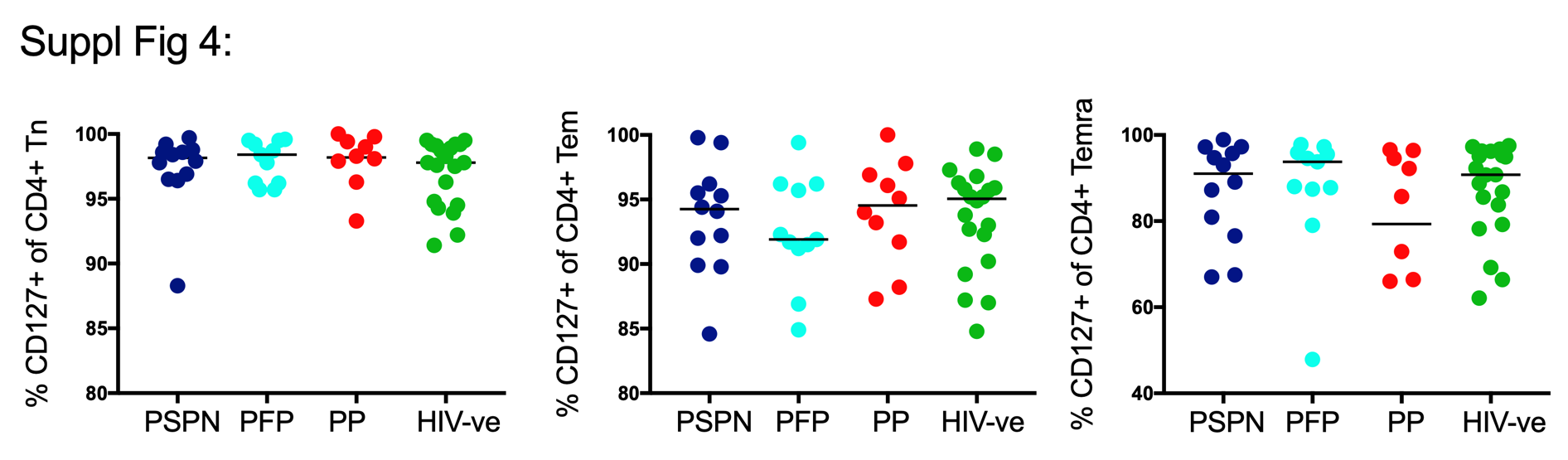

Supplement: Supplementary Figure 4 — Frequency of IL-7R expression on naïve (TN; left), effector memory (EM; middle) and terminally differentiated (Temra; right) CD4 T-cells in PSPN (dark blue; n = 12), PFP (light blue; n = 11), PP (red; n = 10) and uninfected pediatric controls (green; n = 20). For scatterplots, median and interquartile range are shown. Kruskal-Wallis test was performed and corrected for multiple comparisons. [file Image_4.TIFF]

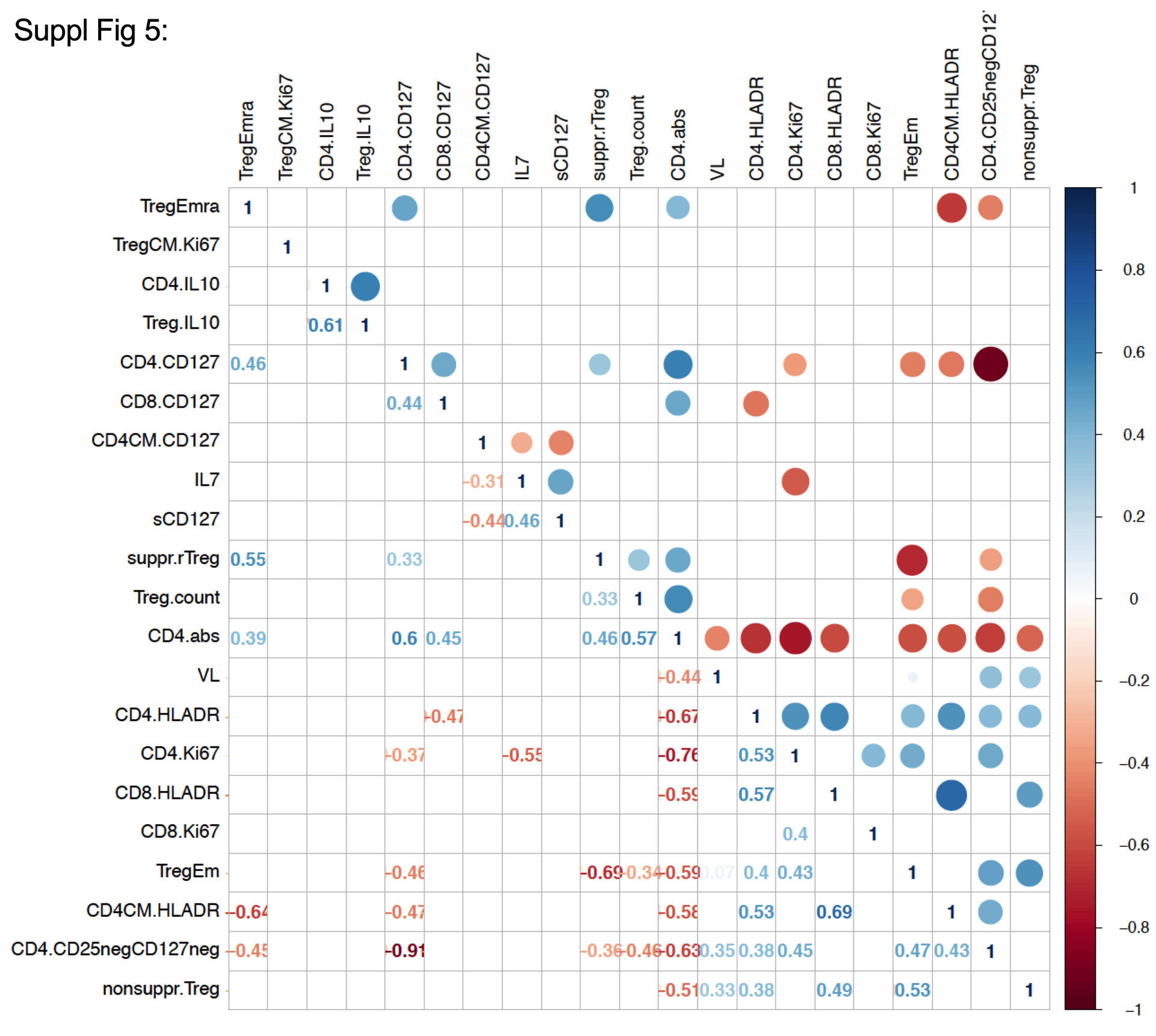

Supplement: Supplementary Figure 5 — Correlation matrix in ART-naïve HIV-infected children (n = 25) with available data for all regarded parameters. Positive correlations are colored in blue and inverse correlations in red with deeper color shading and bigger circle size reflecting stronger r-values. Correlations with p > 0.05 are left blank. [file Image_5.TIFF]
