# Supplementary material for: Increased Regulatory T-Cell Activity and Enhanced T-Cell Homeostatic Signaling in Slow Progressing HIV-infected Children
Source: Front Immunol. 2019 Feb 12;10:213. doi: 10.3389/fimmu.2019.00213 (PMC6379343; doi:10.3389/fimmu.2019.00213)
Supplement: Supplementary Table 1 — List of antibodies for flow cytometry. [file Table_1.pdf]

Suppl table 1:

CD4	BUV 496	SK3	BD
FoxP3	BV 421	PCH101	ebioscience
CD8	V500	RPA-T8	BD
HLA-DR	BV 605	G46-6	BD
CD45RA	BV 650	HI100	BD
CD25	BV 711	BC96	biolegend
Ki-67	BV 785	B56	BD
CD39	AlexaFluor 488	TU66	BD
CCR7	PerCp-Cy5-5	G043H7	biolegend
CD127	PE-Cy5	R34.34	Beckmann Coulter
CD27	PE-Cy7	M-T271	biolegend
CD3	PE-CF594	UGHT1	BD
IL-10	PE	JES3-19F1	biolegend
TGF-β	Alexa Fluor 647	TW4-6H10	biolegend
TNF-α	AlexaFluor 700	Mab11	BD
